# Notes and Notices

**Published:** 1887-09

**Authors:** 


### NOTES AND NOTICES.

Dr. Edward W. Mercer has removed to No. 157 North Fifteenth Street, Philadelphia.

Country Visitors.—The editorial sanctum of this journal has recently been honored by visits from some of our friends. Amongst these we note Drs. Kent, Ballard, Gee, McLaren, etc. The Homœopathic Physician is always glad to welcome its friends, especially those from the *country*.

Wants the Earth.—There is a distinguished physician residing in New Jersey who was recently elected president of his State Society and vice-president (and hence in succession to the presidency) of the I. H. A. He now desires the earth!

Correction.—June number, page 206, seventeenth line from bottom, for gown read *gums*.

The State University of Iowa, Homœopathic Medical Department, has just issued a supplemental announcement from which we copy the following paragraph: "Since the announcements for the coming session were issued, the Board of Regents have appointed Dr. J. G. Gilchrist, professor of surgery, and Dr. C. H. Cogswell, professor of obstetrics and diseases of children, thus establishing what has been long desired, viz.: four full chairs, which include all therapeutic and clinical teaching. The course of instruction as heretofore published is, therefore, modified as follows: instruction in anatomy, physiology, chemistry, and medical jurisprudence will be given in the medical department, while all other instruction, both didactic and clinical, will be given by the faculty of the homœopathic medical department."

Nickel-Plated Cooking-Vessels.—An order has been issued in Lower Austria forbidding manufacturers and tradesmen to sell nickel-plated vessels for cooking. It is stated that vinegar and other substances dissolve nickel, and that this, in doses of the seventh of a grain, produces vomiting, and is generally more poisonous than copper.—*Medical Record.*

The American Institute of Homœopathy held its fortieth session at Saratoga June 27th, 1887. The following officers were elected: President, Dr. A. C. Cowperthwaite, Iowa City; vice-president, Dr. N. Schneider, Cleveland, Ohio; secretary, Dr. Pemberton Dudley, Philadelphia; treasurer, Dr. E. M. Kellogg, New York; provisional secretary, Dr. F. M. Strong, New York. Niagara Falls was selected as the place of meeting in 1888.

Rochester Hahnemannian Society.—This is a society of pure Hahnemannians, few in numbers, but filled with enthusiasm, industry, and perseverance. Its officers are: President, James A. Biegler, M. D.; vice-president, R. A. Adams, M. D.; secretary and treasurer, R. C. Grant, M. D.; censors, A. B. Carr, M. D., Julius Schmitt, M. D., S. George Hermance, M. D.

Bureau of Clinical Medicine, I. H. A.—The chairman of this bureau was erroneously reported in our July number, page 268, to be Dr. H. Hitchcock. This is a mistake. Dr. Alice B. Campbell, of Brooklyn, holds this office.

Sowing Tares.—Dr. T. F. Allen (at one time one of the most enthusiastic apostles of so-called high dilution Homœopathy, which he now admits is unreliable and delusive), in a speech at the alumni dinner of the College of which he is the dean and professor of materia medica, said in substance, in speaking of the College, its hopes and prospects: "It is the aim of the College to make a university, not a medical college where only one branch or department of medicine is taught, but a university where all of medicine is taught. In such a university Homœopathy would be taught, but it would constitute only a part of the course of study. We do not hold that Homœopathy is the exclusive and only law of healing. It is the most widely and generally useful, but not the only guide in the treatment of the sick, and what we want is a medical university where all medicine can be fairly and impartially taught." —*N. Y. Med. Times.*

Taught by an Allopath.—The following is told by a homœopath of St. Louis: He had been treating unsuccessfully a case of porrigo, when, finally, he advised the patient to consult a well-known allopathic dermatologist. Several weeks after he met his former patient entirely cured. In conversation with the dermatological doctor on a subsequent occasion he was curious enough to ask what his treatment had been. "Hepar sulph., internally," was the answer. "Why," said our homœopathic friend, "I gave him Hepar myself." "What trituration?" "Third decimal," was the reply. "Ah!" said the allopath, "you didn't give it high enough; I gave the sixth."—*The Periscope.*

Boxing the Ears.—Dr. Samuel Sexton, of New York city, recently called attention to the injury which frequently follows a blow or box upon the ear. Parents and others should not forget this warning. Dr. Sexton found notes of fifty one cases where the ear had been injured by blows. The Doctor thinks many cases of ear troubles are so caused, which are never traced to blows, etc. If a child need be whipped, don't overlook the old-fashioned slipper and buttock operation!

Salicylic Acid in Foods.—There is a growing belief among sanitarians that salicylic acid is being used more and more extensively in the preservation of canned foods, milk, wine, beer, and other articles. To such an extent was this done in Paris that the French Government has already twice taken action upon the matter. Dr. E. H. Bartley, Chemist to the Brooklyn Health Department, has recently called attention to this matter. He states that in 1885 the chief adulterations which he found in beer were yeast and bicarbonate of soda. Lately he has examined several different kinds of bottled beer sold in Brooklyn, the list including some of the Western beers. He has found salicylic acid in them. The amount of this acid required to preserve beer is about twelve to fifteen grains per gallon. Salicylic acid, if taken continuously, tends to injure digestion and irritate the kidneys.—*Med. Record.*

Diet in Hypochondriasis.—An excessive meat diet will sometimes bring on hypochondriasis, and in this condition the ordinary rules for nervous invalids are to be changed. Hypochondriacs must be fed largely upon vegetable food, which distends the colon and causes it to empty itself. When hypochondriasis is brought on by a meat diet, it is cured by porridge and green vegetables.—*C. E. Dana.*

Epidemic Remedies with an Antipsoric.—When a remedy is found to be the homœopathic specific for a reigning epidemic of intermittent fevers [probably the same applies to other diseases.—Eds.], and there is, notwithstanding, now and then, a patient whom it does not cure in a perfect manner, and no influence of marshy country opposes its operation, then the obstacle generally arises from the psoric miasm, and, consequently, antipsoric medicines ought to be employed until perfect health is restored.—*Hahnemann.*

Undeveloped Symptoms.—A case that is very rare in chronic diseases, but which is sometimes met with in acute, is one where, notwithstanding the indistinctness of the symptoms, the patient feels himself very ill; which may be ascribed to the depressed state of sensibility that does not permit him to have a clear conception of his symptoms. In a case of this nature Opium (in a high potency) will remove the torpor of the nervous system, and then the symptoms of the disease will develop themselves plainly in the reaction of the organism.—*Hahnemann.*

Bad Breath.—Dr. Frank H. Gardiner believes that decaying particles in the mouth as far back as the pharynx vault taint the breath exhaled very little, if at all. Mouth-breathers have a bad breath when the tonsils are enlarged, or when cheesy masses exist in the tonsillary mucous folds. Certain gastric derangements taint the breath only when gases are eructated through the mouth. The principal cause of bad breath is decomposition in the intestinal canal, the retention of fecal matters in the transverse and descending colon, and the absorption of gases into the circulation, finally exhaled by the lungs. Catarrh (nasal, pharyngeal, laryngeal, or bronchial) causes bad breath. This bad breath is often a source of serious annoyance to patients, and the fact that it has more than a local cause is too often ignored by the physician, who, therefore, fails to cure it.—*Dental Review.*

THE PULSE.—Speaking of lectures upon the pulse by a Dr. Broadbent, *The Lancet* wrote: "One has only to turn to the writings of Hippocrates to learn how closely and how accurately its (the pulse's) variations were observed, with much remarkable result in prognosis and treatment." Alas for the uncertainties of historical reminiscence! Hippocrates was a great physician, and it is generally thought safe to credit him with first knowing, in a crude way, of course, a little of every part of our great art. But Hippocrates, in fact, knew nothing of the pulse, wrote nothing about it, and quite neglected that time-honored source of professional information. Herophilus was one of the first to investigate it, while Galen erected its study into a separate science, and fixed irrevocably upon the profession the practice of fingering the wrist as part of every medical service.—*Med. Record.*

TO ARREST NASAL HEMORRHAGE.—In persistent hemorrhage from the nasal cavity, plugging the posterior nares should not be done until an attempt has been made to check the hemorrhage by firmly grasping the nose with the finger and thumb, so as completely to prevent any air from passing through the cavity in the act of breathing. This simple means, if persistently tried, will, in many cases, arrest the bleeding. The hemorrhage persists because the clot which forms at the rupture in the blood-vessel is displaced by the air being drawn forcibly through the cavity in the attempt of the patient to clear the nostrils. If this air is prevented from passing through the cavity, the clot consolidates in position and the hemorrhage is checked.—*Edinburgh Medical Journal.*

DEATH FROM POISON EJECTED BY A TOAD.—A child æt. six years, while throwing stones at a toad, suddenly felt the animal spurt some moisture into his eye. There suddenly set in slight pain and spasmodic twitching of the injected eye; two hours after coma, jumping sight, desire to bite, dread of food and drink, constipation, abundant urine, and great agitation manifested themselves, followed on the sixth day by sickness and apathy and a kind of stupor, but with a regular pulse. Some days later, having become comparatively quiet, he left his bed; his eyes were injected, the skin dry, the pulse free from fever.

He howled and behaved like a madman, sank into imbecility and speechlessness, from which he never rallied.—*Druggist and Chemist, Phila.*

CANTHARIDES FOR HYDROPHOBIA.—A Russian doctor says that he has successfully treated with Cantharides some patients who were bitten by a rabid wolf. Three men were badly bitten by the animal in various parts of the body, and Cantharides plasters were applied to the wounds. At the same time powdered Cantharides was administered to each in doses of one grain each day, until certain well-known symptoms were exhibited. These patients have now been in perfect health for eight months since the bites were given, and it is hoped that Cantharides has thus proved a successful remedy to the dire disease with which they were threatened.—*Chambers' Journal.*

"The world do move"—but very slowly! This use of the *Spanish Fly* has been laid down in the homœopathic works since the first provings in 1802 by Hahnemann and others, and what is more, has proven successful in many cases of hydrophobia. Pasteur's discoveries of the virtues of inoculation by the virus of the mad dog, reduced in action by passing through several of the lower animals, which discoveries have been heralded over the civilized world, were anticipated years ago by Dr. J. B. Cox's provings of the same virus in Philadelphia. All large homœpathic text-books detail the uses of this poison—*Hydrophobinum* or *Lyssin.* "The truth is mighty and will prevail."—*Minnesota Medical Monthly.*

